# Clustering probabilistic tractograms using independent component analysis applied to the thalamus

**DOI:** 10.1016/j.neuroimage.2010.09.054

**Published:** 2011-02-01

**Authors:** Jonathan O'Muircheartaigh, Christian Vollmar, Catherine Traynor, Gareth J. Barker, Veena Kumari, Mark R. Symms, Pam Thompson, John S. Duncan, Matthias J. Koepp, Mark P. Richardson

**Affiliations:** a Department of Clinical Neuroscience, Institute of Psychiatry, King's College London, London, UK; b National Society for Epilepsy MRI Unit, Dept. of Clinical and Experimental Epilepsy, UCL Institute of Neurology, Queen Square, London WC1N 3BG, UK; c Department of Neuroimaging, Institute of Psychiatry, King's College London, London, UK; d Department of Psychology, Institute of Psychiatry, King's College London, London, UK

## Abstract

The connectivity information contained in diffusion tensor imaging (DTI) has previously been used to parcellate cortical and subcortical regions based on their connectivity profiles. The aim of the current study is to investigate the utility of a novel approach to connectivity based parcellation of the thalamus using probabilistic tractography and independent component analysis (ICA). We use ICA to identify spatially coherent tractograms as well as their underlying seed regions, in a single step. We compare this to seed-based tractography results and to an established and reliable approach to parcellating the thalamus based on the dominant cortical connection from each thalamic voxel (Behrens et al., 2003a,b). The ICA approach identifies thalamo-cortical pathways that correspond to known anatomical connections, as well as parcellating the underlying thalamus in a spatially similar way to the connectivity based parcellation. We believe that the use of such a multivariate method to interpret the complex datasets created by probabilistic tractography may be better suited than other approaches to parcellating brain regions.

## Research Highlights

► Independent component analysis used to segment the thalamus ► Promising technique to parcel out consistent fibre tracts ► Independent components consistent with tractography from the same region ► Independent components consistent with other clustering methods ► Multivariate method applied to segment probabilistic tractography

## Introduction

Parcellation of the thalamus based on magnetic resonance imaging (MRI) derived measures of connectivity is a robust, reproducible technique, which makes use of the information extractable from diffusion tensor imaging (DTI) data by tractography techniques (Behrens et al., 2003a; Johansen-Berg et al., 2004; Traynor et al., 2010). It represents a promising approach to better localise targets for clinical methods such as deep brain stimulation. Although the results are highly reproducible (Traynor et al., 2010), and the regions defined appear functionally relevant (Johansen-Berg et al., 2004), the method implicitly models only long range thalamic connections and the results therefore cannot constitute a full description of the thalamus. Further work is needed to fully elucidate the relationship of the MRI derived thalamic parcellation to the cytoarchetectonic descriptions of the Morel atlas. (Preliminary data from our own group suggests that correspondence to may not be as high as might initially be expected; a result which is perhaps not surprising given that the atlas is concerned with local histological anatomy, rather than remote connections of individual nuclei). Overall, the thalamus continues to be somewhat neglected in human neuroimaging, and its substructure and connections are not well described *in vivo*.

In addition to studies of the thalamus, probabilistic tractography has been used to parcellate different regions of cortex based on connectivity profiles. The most popular method has been using spectral clustering to separate out different cortical regions based on their spatial pattern of connectivity using some similarity measure, most commonly spatial correlation (Anwander et al., 2007; Beckmann et al., 2009; Johansen-Berg et al., 2004; Klein et al., 2007). Unfortunately, as originally implemented, these methods require as input the number of clusters hypothesised (a-priori) to exist in the dataset, a specification of to where these clusters project, or both, and are therefore not wholly user independent. More recently, a number of promising approaches to obviate the need for these constraints, such as the use of Dirichlet process priors, have been reported to segment streamline tractography (Wang et al., 2009) and parcellate subcortical structures based on probabilistic tractography (Jbabdi et al., 2009).

Independent component analysis (ICA) is a blind source separation technique whereby independent “sources” which have been mixed together are separated by maximising independence between them (Calhoun et al., 2001). As a method, it has enjoyed significant success in MR research, especially in functional MRI (fMRI). ICA has been shown to reproducibly detect functional networks both within and across subjects (Beckmann et al., 2005) and the resulting networks in fMRI have been shown to correspond to functional networks (Smith et al., 2009).

There are few published papers applying ICA to diffusion tensor (DTI) data. After establishing the non-Gaussian nature of the diffusion signal, Singh et al. (2006) demonstrated the utility of ICA in recovering multiple tensors in voxels where crossing fibres are likely to occur. Arfanakis et al. (2002) used spatial ICA to extract components related to eddy-current induced artefact. Although a component was also found that appeared related to the structure of the internal capsule, no further analysis was performed. In both cases, ICA was used to maximise ability to detect the tracts or improve data quality, not to investigate the tracts themselves. Following the principal of independence between components, we propose that ICA could be an effective method to blindly segment anatomical tracts of interest without the inherent bias of region-of-interest based approaches (Catani et al., 2002; Wakana et al., 2004). It may also allow the full use of the very large amount of information contained in probabilistic tractograms (Robinson et al., 2008).

The lack of prior use of ICA for tractography purposes probably lies in computational demands. With the increasing availability of 64-bit operating systems, however, limits to dataset size based on the amount of random-access-memory available are becoming less problematic and processing power continues to grow. Moreover, as the number of papers investigating clustering in DTI attest (e.g. Anwander et al., 2007; Beckmann et al., 2009; Jbabdi et al., 2009; Klein et al., 2007), it is timely to examine ICA in this context.

The aim of this study is to investigate the use of ICA for clustering DTI tractography data. In particular, we examined its use in segmenting the thalamus, comparing the results to an analysis of the same data using the method from Behrens et al. (2003a).

## Methods

### Subjects

This study was approved by the King's College Hospital Research Ethics Committee (Ethics Ref 06/Q0703/124). Thirty-eight healthy controls were recruited via a local volunteer's database (21 female, mean age 31.74 years, range 23–52 years, standard deviation 6.71 years). These DTI datasets were collected as part of a larger study. After a full explanation of the methods involved, each subject gave written informed consent to take part in the study.

Each participant was scanned using a GE Signa 3 T HDx system (General Electric, Waukshua, WI, USA), with actively shielded magnetic field gradients (maximum amplitude 40 mT m^−1^). Each volume of DTI data was acquired using a cardiac-gated, multi-slice peripherally-gated doubly refocused spin echo EPI sequence, optimised for precise measurement of the diffusion tensor in parenchyma, from 60 contiguous near-axial slice locations with isotropic (2.4 × 2.4 × 2.4 mm) voxels. The echo time was 104.5 ms while the effective repetition time varied between subjects in the range 12 and 20 RR intervals and an ASSET speed up factor of 2 was used. Thirty-two images were collected at a diffusion of 1300 s mm^−2^–with gradient directions uniformly distributed in space–together with 4 images acquired with no diffusion gradients applied.

## Study 1: ICA analysis of probabilistic tractograms

### Creation of FA maps and alignment to a common space

**Fig. 1:**
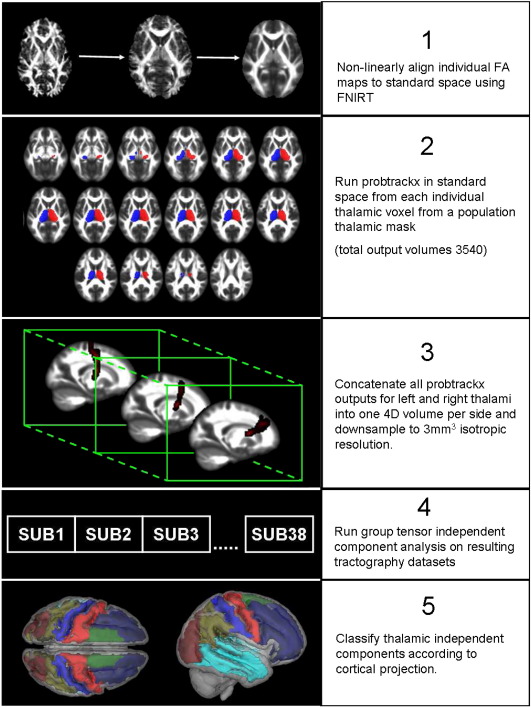
Processing steps for independent component analysis on tractography data. See figure text for details.

Diffusion weighted images were corrected for the effects of eddy-current induced distortion, and subject motion, using in-house software (Jones et al., 2002) (see Fig. 1). Fractional Anistropy (FA) images were created by fitting the diffusion tensor to this eddy-corrected, masked data using dtifit (part of the FMRIB diffusion toolkit, http://www.fmrib.ox.ac.uk/fsl/fdt/fdt_dtifit.html). All subjects' FA data were then aligned into a common space (FSL's standard FA template, in approximate MNI space) using the nonlinear registration tool FNIRT (Andersson et al., 2007). The best way to register diffusion weighted images is a subject of ongoing research, and is dependent on the type of analysis to be performed on the resulting images; the registered FA images were therefore visually inspected alongside the FA template for any evidence of registration abnormalities.

### Tractography and concatenation

Probabilistic tractography was carried out using bedpostX/probtrackX (Behrens et al., 2003b), part of the FSL toolkit. Bedpostx uses Monte Carlo Markov chain sampling to estimate the diffusion parameters at each voxel and also calculates the necessary parameters for probabilistic tractography. In this case up to 2 fibres were modelled per voxel. A burn-in of 5000 was chosen instead of the default value of 1000 to ensure convergence of the Markov chains, from which the posterior distributions of the local estimate of the fibre orientation distribution were sampled.

Following bedpostX, probtrackx was run on each individual thalamic voxel. Probtrackx repeatedly samples from the voxel-wise principal diffusion direction calculated in bedpostx, creating a new streamline in each iteration. This builds a distribution on the likely tract location and path, given the data. The thalamus mask used was taken from the Harvard-Oxford subcortical atlas and was specified in MNI space. To ensure all thalamic voxels across subjects were incorporated, the thalamic mask was dilated by two voxels. Tractograms were therefore calculated in an approximate MNI space, using the normalisation parameters calculated from FNIRT.

In this processing step, 5000 streamlines were followed from each seed voxel, resulting in a single 3D volume “tractogram” per seed voxel. As only thalamo-cortical connections were of interest, the brainstem was used as an exclusion mask to avoid thalamo-spinal fibres. A further exclusion mask was specified on the midline to reject artefactual fibres crossing the corpus callosum. The resulting 1770 tractograms were then concatenated into two 4D images (one 3D tractogram per seed voxel), representing right and left thalamic seeds, respectively. Finally, in order to reduce computation load in subsequent processing steps, these 4D images were downsampled to a 3-mm^3^ isotropic voxel size in their spatial dimension.

### ICA data analysis

The ICA algorithm melodic (part of the FSL toolkit) was used to identify independent components across subjects. The tensorial extension of ICA (Beckmann and Smith, 2005) was used, which adds “subject” to the space and time domains commonly used in ICA for fMRI, to allow investigations of groups. In practice, this means that both the temporal and the spatial aspect of the components are constrained to be consistent across subjects. In the context of this experiment, each volume in the 4D image represents the probabilistic tractogram of one seed voxel (from each thalamic voxel, calculated above), and is analogous to a functional timeseries in which each volume reflects time varying functional responses. Specifically, this means that, in the analyses presented here, the output of melodic will be spatial components with common underlying seed regions across subjects, assumed to be clusters of fibre pathways. Each spatial independent component found by melodic was thresholded using mixture modelling (Beckmann et al., 2003), with a Gaussian distribution used to model background noise and a single gamma distribution used to model signal of interest, in this case modelling the positive tail of the distribution. The gamma distribution was thresholded at a posterior probability of *p* > 0.5, a balance where the chance of false positive is equal to the chance of false negative (Woolrich et al., 2005).

The contribution of each tractogram (and simultaneously the voxel from which the tractogram is calculated) to each independent component was converted into a normalised *Z* statistic, calculated as the distance of a particular tractogram's representation from the mean representation of all tractograms in units of standard deviation. This allows the localisation of the seed voxels that contribute towards an independent component, in this case a presumed white matter pathway, as well as the quantification of the size of the effect of their contribution. This method assumes that thalamic clusters are distributed similarly across the healthy controls tested (which is tested in study 2). This statistic was thresholded for each component at 3.1, representing a *p*-value of less than 0.001 that a thalamic voxel contributes towards an individual component.

### Post-hoc anatomical classification

Components projecting from the thalamus were classified according to whether they passed through one of seven cortical regions of interest (extracted in standard space using the Harvard-Oxford cortical atlas provided as part of FSL 4.1.5); any component that passed through two cortical ROIs was classified as contributing to both. The regions of interest chosen were the frontal lobes, supplementary motor area (SMA), precentral gyrus, postcentral gyrus, parietal lobes, occipital lobes and temporal lobes and correspond to the regions chosen in Behrens et al. (2003a).

## Study 2: correspondence between independent components and “Raw” tractography

Voxels identified in the previous analysis as significantly contributing towards a particular independent component were used as seeds to assess correspondence between the tractograms estimated using ICA and tractography directly estimated from the same voxels.

Tractograms from seeds identified in the previous analysis were summed within subjects, binarised, and then summed across subjects. The resulting population tractogram was thresholded at three different levels such that only regions occurring in 25%, 50% and 75% of subjects were included. To assess the overlap between these tractograms and their corresponding independent components, the Dice coefficient was used; for a pair of images (*X* and *Y*) this is given by $\frac{\left|X\cap Y\right|}{\left|X\right|+\left|Y\right|}$ where, in this case, *X* and *Y* represent the number of voxels in the thresholded independent component (*X*) and the thresholded population tractography image (*Y*), respectively.

To assess the sensitivity of the ICA method relative to the “ground truth” (in this case, the results of the raw tractography), the area of overlap, calculated above, was divided by the total volume of tractography results (at the three thresholds specified above) to give the proportion of the tractogram detected by ICA.

## Study 3: correspondence between ICA and target based classification for parcellating the thalamus

The classification method described by Behrens et al. (2003a) follows a “winner-takes-all” approach. In the work described here, probtrackx was run using a mask of the left and right thalamus (as used in the ICA analysis) and the same 7 cortical classification targets were used to classify output components in the ICA analysis. 5000 streamlines were followed from each voxel in the thalamus mask to each target region. The outputs produced for each target region were then combined using find_the_biggest (part of the FSL toolkit). This process involved classifying each voxel in the thalamus depending on the target to which the greatest number of the paths from that voxel propagated (winner-takes-all). The same subjects were used as in the ICA based analysis.

To create a hard-segmentation map representative of the group, the individual binarised thalamus classifications for each region were concatenated and a one-sample *t*-test was assessed by permutation using randomise (again, part of the FSL toolkit). This was repeated for all 7 cortical target regions. Thalamic voxels with an uncorrected *p*-value of *p* < 0.001 were classified as being associated with the cortical target.

Using the post-hoc anatomical classifications made in study 1, thalamic voxels were classified as being associated with the same 7 cortical regions. The normalised *Z* scores calculated for at each thalamic voxel for each independent component were also thresholded at an uncorrected *p*-value of *p* < 0.001 (*Z* > 3.09). Clusters associated with each of the 7 cortical regions were then summed and binarised. As in study 2, the Dice coefficient was used to measure overlap between the two segmentation methods.

## Results

### Study 1

**Fig. 2:**
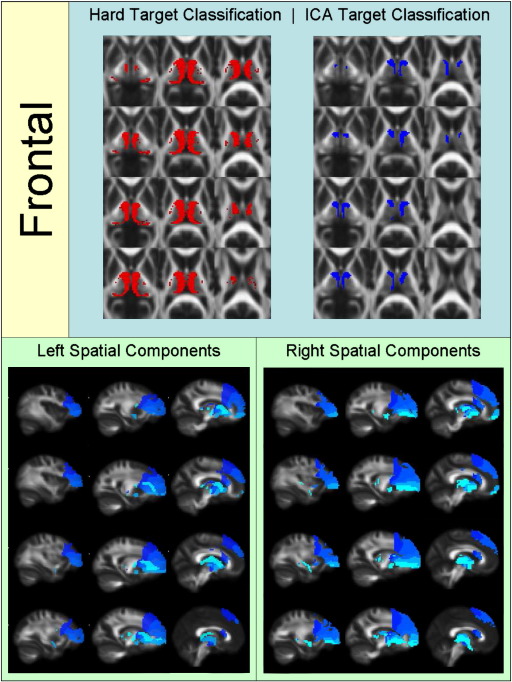
Regions of the thalamus significantly connected to frontal cortex in 38 healthy controls using the Behren's hard segmentation method (top left) and using ICA (top right). ICA segmentation represents the sum of independent components projecting to frontal cortex (bottom left and right). Different shades of blue represent different independent components (this also applies to Figs. 3–8).

**Fig. 3:**
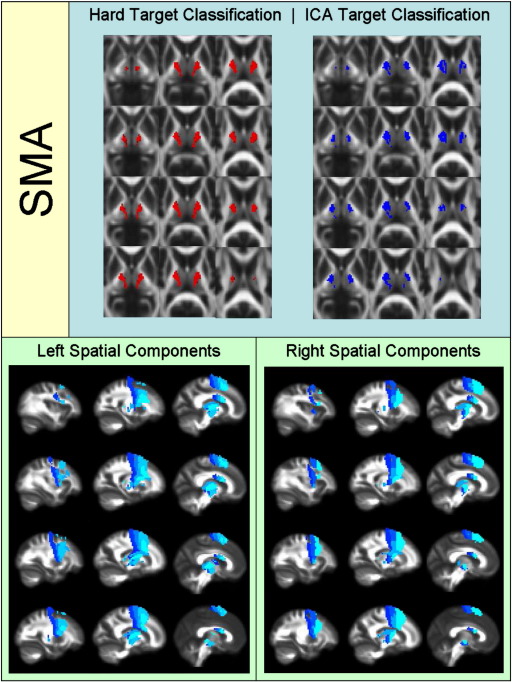
Regions of the thalamus significantly connected to supplementary motor cortex.

**Fig. 4:**
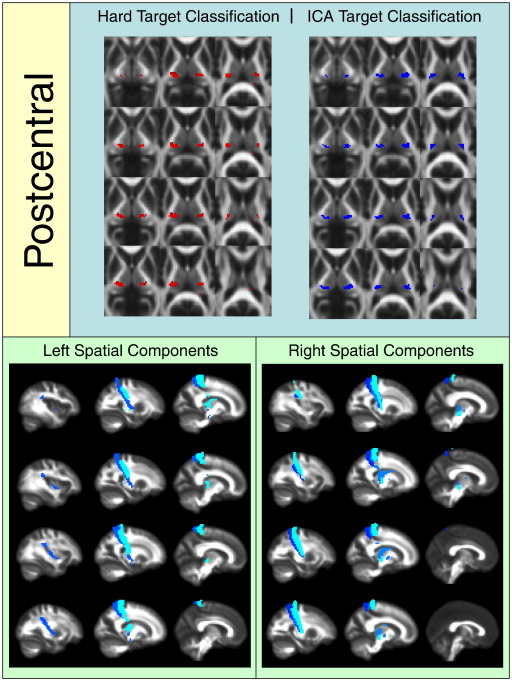
Regions of the thalamus significantly connected to the precentral gyrus.

**Fig. 5:**
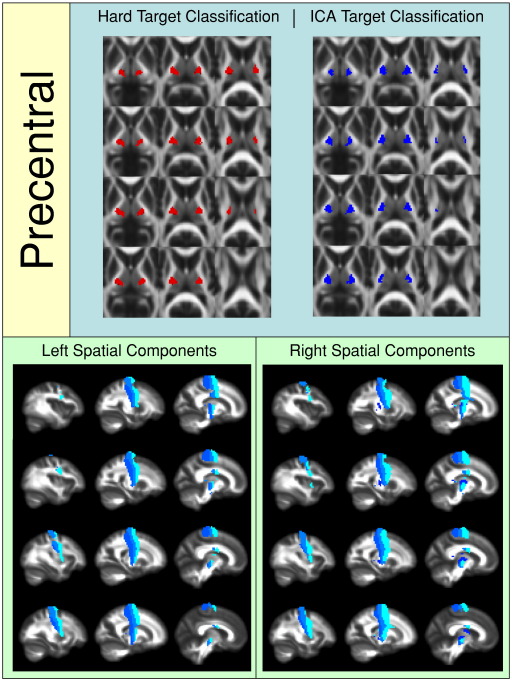
Regions of the thalamus significantly connected to the postcentral gyrus.

**Fig. 6:**
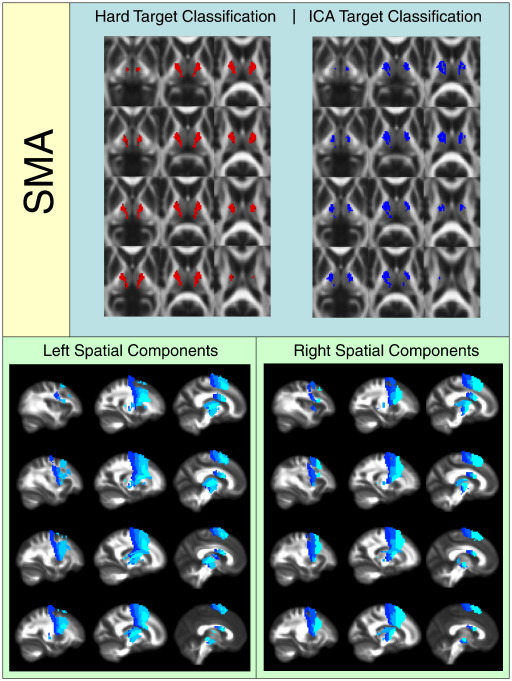
Regions of the thalamus significantly connected to parietal cortex.

**Fig. 7:**
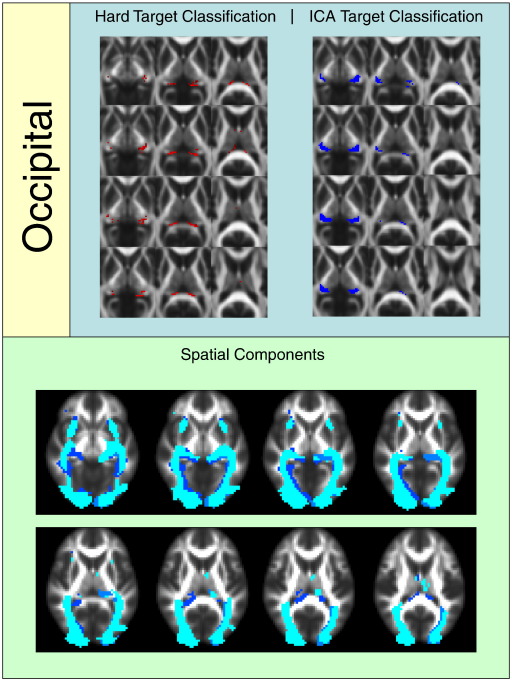
Regions of the thalamus significantly connected to occipital cortex.

**Fig. 8:**
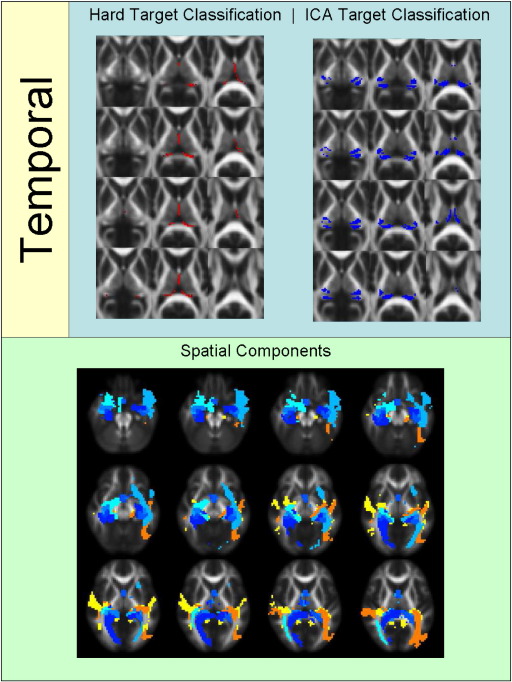
Regions of the thalamus significantly connected to temporal cortex. Different shades of blue represent different independent components. Shades of yellow/orange show components corresponding to the acoustic radiations. Note that these components also include fibres projecting to posterior parietal cortex.

Figs. 2 to 8 show regions of the thalamus classified according to the Behrens method (top left of each figure), ICA based classification (top right of each figure), as well as the resulting independent components per hemisphere. Supplementary Tables 1 and 2 shows the projections of each component per thalamus. The components are anatomically plausible; for example components representing the optic radiation can be seen in Fig. 7, and components consistent with the acoustic radiation are present in Fig. 8.

### Study 2: overlap between independent components and raw tractography

**Fig. 9:**
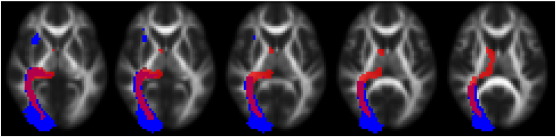
Overlap between occipital independent component (blue) and the sum of the binarised tractograms (red). The red component represents overlap between at least 50% of subjects (19 of 38).

Table 1 shows the mean Dice coefficients for each component and resulting between-subjects tractogram per gross cortical target ROI (see Supplementary Tables 1 and 2 for component-by-component coefficients). Fig. 9 shows the overlap between an example component (in this case corresponding to the optic radiation) and the raw tractography from the seed voxels identified from this component (right component 6 in Supplementary Table 1). At low population thresholds, overlap ranges from 0.3–0.5 decreasing linearly to 0.2–0.4 at higher population thresholds.

Table 2 shows the proportion of volume of overlap between ICA and raw tractography (calculated above) as a function of the population tractograms at different thresholds. As the population threshold increases, so does the proportion of raw population tractography data accounted for by the independent component.

### Study 3: overlap between ICA and target-based classification

As is clear from the figures and from the overlap ratios below (Table 3) and region-by-region analyses shown in Figs. 2–8, correspondence between the two methods is quite good in precentral, postcentral and SMA regions (overlap > 0.5).

Occipital and temporal classification results showed incomplete overlap. Looking at Figs. 7 and 8, the ICA based method showed larger thalamic regions of occipital and temporal connectivity. However, this may show insensitivity or large group variability on the part of the hard segmentation method. The identified spatial components show anatomically plausible segments of the optic radiation bilaterally (Fig. 7) and of the acoustic radiation (Fig. 8, yellow/orange components).

Frontal (Fig. 2) and parietal (Fig. 6) clusters also showed poor overlap between the two methods. The thalamic regions identified using the hard segmentation method were in both cases significantly larger than the corresponding ICA segmented methods. These two observations emphasise that the hard segmentation method is biased towards the dominant connectivity of a voxel.

## Discussion

The use of ICA to segment probabilistic tractograms, as implemented here, represents a novel approach to dealing with the multivariate connectional information available in DTI. The independent components illustrated here spatially overlap with tractography seeded from the same regions. However, in some regions this overlap is small.

This level of overlap is perhaps not surprising given the inherent differences in approaches. Whereas probabilistic tractography outputs a spatial map of any connectivity from a given seed region, ICA in this context outputs a map of projections/streamline *patterns* that are spatially consistent across different seed voxels. Nonetheless, the ICA approach appears to well represent dominant connections from thalamic regions. This is especially clear at high population tractography thresholds, where > 80% of the volume of tractograms is present in the related IC, though the Dice coefficient may be less than 0.5.

**Fig. 10:**
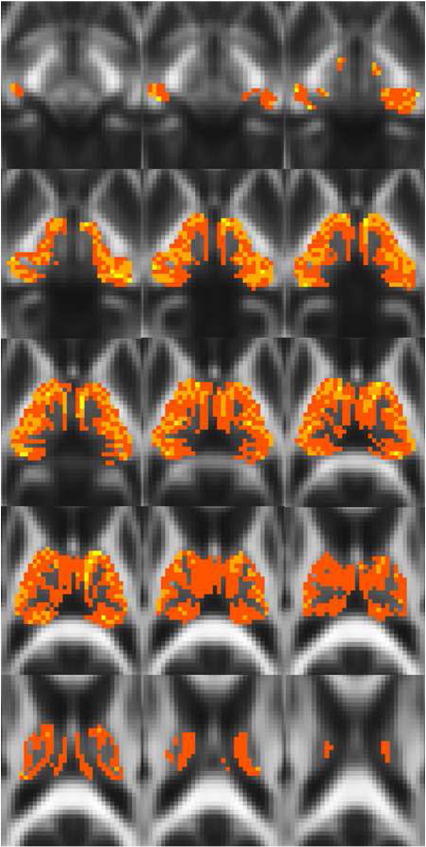
Density of independent component clusters across both thalami. Yellow indicates that a voxel is a seed for 3 components, light orange 2 and orange 1.

The resulting ICA-based segmentation of the thalamus overlaps spatially with the implementation of Behrens et al. (2003a) hard segmentation method in the same datasets. The results of the hard segmentation method are also visually similar to those reported by Behrens and colleagues. However, the ICA-derived clusters are more conservative for frontal and parietal regions and less conservative for occipital and temporal regions. This may be a function of one of the clear advantages of the ICA method; there is no constraint that individual seed voxels should be involved in just one cluster / component (see Fig. 10). This is more realistic in the context of standard DTI resolution of ~ 2 mm^3^, where multiple white matter pathways could easily reside within the same unit voxel. In some of the temporal components bilaterally (Fig. 8), occipital projections are evident and vice versa (Fig. 7), indicating spatial overlap between these regions at this resolution. A hard segmentation method is unlikely to be sensitive to this. Moreover, the segmentation results are consistent bilaterally, even though the left and right thalami were analysed independently.

The new method presented here leads to different approach to assessing connectivity compared to standard ROI-based tractography. The output of this type of ICA analysis leads to spatial maps that characterise not just tractograms, but also their seed regions. In the context of the emerging field of connectonomics (Sporns et al., 2005), such an approach allows an unbiased method to creating meaningful ROIs for further analysis using other techniques, such as fMRI, in place of using population atlases. This is computationally demanding. ICA analysis of each individual thalamus needed a minimum 8 GB of RAM. Up-scaling to a full cortical analysis would need approximately 128 GB (at the same resolution). Nonetheless, as computer power and memory becomes cheaper, this method becomes more feasible.

Another approach to reducing dimensionality of probabilistic tractography is by creating anatomical connectivity maps (Stamatakis et al., 2009), which parameterise connectivity in each voxel by summing the number of probabilistic streamlines that pass through that voxel from every other voxel. Although this approach attempts to incorporate connectivity information, it ignores the spatial distribution of the individual tractograms from which it is derived. The method demonstrated here allows the use of the full voxel-by-voxel distribution of connectional information contained in probabilistic tractograms, using a multivariate analysis method to interpret the probabilistic tractography data available.

## Figures and Tables

**Table 1:** Average Dice overlap values between tractograms (thresholded at 25%, 50% and 75% overlap between subjects) and independent components per gross cortical target.

		25%	50%	75%
Left	Frontal	0.409	0.318	0.235
Temporal	0.374	0.316	0.258
Occipital	0.312	0.239	0.172
Parietal	0.318	0.256	0.189
SMA	0.448	0.388	0.304
Postcentral	0.437	0.391	0.315
Precentral	0.548	0.496	0.406
Caudate	0.437	0.437	0.386
Right	Frontal	0.405	0.322	0.246
Temporal	0.343	0.272	0.209
Occipital	0.333	0.250	0.183
Parietal	0.352	0.281	0.214
SMA	0.421	0.363	0.289
Postcentral	0.544	0.509	0.433
Precentral	0.483	0.429	0.341
Caudate	0.403	0.371	0.314

**Table 2:** Proportion of volume of overlap between tractograms and independent components represented by tractograms.

		25%	50%	75%
Left	Frontal	0.672	0.714	0.756
Temporal	0.613	0.692	0.772
Occipital	0.574	0.634	0.679
Parietal	0.656	0.734	0.806
SMA	0.682	0.770	0.848
Postcentral	0.703	0.801	0.886
Precentral	0.712	0.794	0.880
Caudate	0.444	0.566	0.691
Right	Frontal	0.680	0.735	0.791
Temporal	0.626	0.691	0.747
Occipital	0.667	0.732	0.776
Parietal	0.686	0.772	0.845
SMA	0.666	0.763	0.850
Postcentral	0.650	0.751	0.833
Precentral	0.670	0.771	0.862
Caudate	0.445	0.548	0.685

**Table 3:** Dice coefficient values between thalamic clusters identified using ICA and hard segmentation.

	Left	Right
Region	Overlap	
Frontal	0.394	0.404
Occipital	0.124	0.511
Parietal	0.386	0.480
Postcentral	0.772	0.713
Precentral	0.707	0.766
Premotor	0.703	0.679
Temporal	0.148	0.218
