# Supplementary material for: Clustering probabilistic tractograms using independent component analysis applied to the thalamus
Source: Neuroimage. 2011 Feb 1;54(3):2020–32. doi: 10.1016/j.neuroimage.2010.09.054 (PMC3032893; doi:10.1016/j.neuroimage.2010.09.054)
Supplement: Supplementary file 1 — Supplementary Materials. [file mmc1.doc]

Supplementary Table 1: Dice coefficient and tractography overlap of each individual independent component in the left thalamus with the raw tractogram from the ICA defined seed region.

| **Left Thalamus** | | | | | | | | |
| --- | --- | --- | --- | --- | --- | --- | --- | --- |
| *IC* | *Dice 25%* | *Tractography % of overlap* | *Dice 50%* | *Tractography % of overlap* | *Dice 75%* | *Tractography % of overlap* | *Primary Projection* | *Secondary Projection* |
| 1 | 0.332 | 66.7% | 0.245 | 74.6% | 0.175 | 79.7% | Occipital |  |
| 2 | 0.360 | 74.4% | 0.276 | 78.4% | 0.197 | 80.7% | Frontal |  |
| 3 | 0.302 | 74.4% | 0.224 | 79.9% | 0.159 | 83.2% | Parietal | Temporal |
| 4 | 0.413 | 69.8% | 0.311 | 72.6% | 0.223 | 73.3% | Frontal |  |
| 5 | 0.315 | 52.5% | 0.250 | 56.5% | 0.183 | 59.6% | Occipital |  |
| 6 | 0.289 | 53.2% | 0.223 | 59.1% | 0.159 | 64.4% | Occipital |  |
| 7 | 0.317 | 73.2% | 0.253 | 81.0% | 0.190 | 86.8% | Parietal | Temporal |
| 8 | 0.380 | 71.4% | 0.319 | 78.1% | 0.246 | 85.7% | SMA |  |
| 9 | 0.324 | 69.7% | 0.278 | 78.8% | 0.210 | 86.6% | Parietal | Postcentral |
| 10 | 0.445 | 73.3% | 0.350 | 74.8% | 0.251 | 75.5% | Frontal |  |
| 11 | 0.452 | 67.7% | 0.403 | 79.1% | 0.325 | 84.4% | SMA |  |
| 12 | 0.457 | 72.7% | 0.409 | 80.5% | 0.327 | 87.1% | SMA | Precentral |
| 13 | 0.573 | 75.9% | 0.508 | 83.4% | 0.402 | 92.0% | Precentral |  |
| 14 | 0.410 | 69.9% | 0.329 | 74.4% | 0.236 | 79.2% | Temporal |  |
| 15 | 0.397 | 74.9% | 0.341 | 82.0% | 0.270 | 90.0% | Postcental |  |
| 16 | 0.646 | 71.6% | 0.595 | 78.7% | 0.511 | 90.1% | Precentral |  |
| 17 | 0.423 | 55.2% | 0.372 | 64.5% | 0.309 | 72.4% | Temporal |  |
| 18 | 0.431 | 50.9% | 0.390 | 61.8% | 0.340 | 70.6% | Temporal |  |
| 19 | 0.475 | 69.7% | 0.367 | 74.2% | 0.271 | 80.1% | Frontal |  |
| 20 | 0.496 | 52.9% | 0.458 | 60.2% | 0.408 | 67.6% | Temporal |  |
| 21 | 0.324 | 62.0% | 0.244 | 70.7% | 0.174 | 82.1% | SMA |  |
| 22 | 0.354 | 48.8% | 0.287 | 56.9% | 0.233 | 68.4% | Frontal |  |
| 23 | 0.273 | 52.5% | 0.201 | 62.8% | 0.143 | 80.6% | Temporal |  |
| 24 | 0.330 | 45.2% | 0.267 | 53.7% | 0.198 | 65.8% | Parietal |  |
| 25 | 0.590 | 66.2% | 0.555 | 79.5% | 0.464 | 89.1% | Postcentral |  |
| 26 | 0.515 | 64.6% | 0.474 | 74.8% | 0.382 | 82.7% | SMA | Precentral |
| 27 | 0.421 | 37.6% | 0.449 | 54.3% | 0.391 | 73.1% | DRC* |  |
| 28 | 0.464 | 55.1% | 0.440 | 66.8% | 0.351 | 73.0% | DRC* |  |
| 29 | 0.559 | 70.6% | 0.479 | 78.6% | 0.369 | 87.0% | SMA |  |
| 30 | 0.368 | 32.0% | 0.400 | 45.3% | 0.393 | 62.8% | DRC* |  |

*DRC indicates that the component does not reach a cortical target

Supplementary Table 2: Dice coefficient and tractography overlap of each individual independent component in the right thalamus with the raw tractogram from the ICA defined seed region.

| **Right Thalamus** | | | | | | | | |
| --- | --- | --- | --- | --- | --- | --- | --- | --- |
| *IC* | *Dice 25%* | *Tractography % of overlap* | *Dice 50%* | *Tractography % of overlap* | *Dice 75%* | *Tractography % of overlap* | *Primary Projection* | *Secondary Projection* |
| 1 | 0.347 | 70.9% | 0.266 | 75.7% | 0.200 | 80.4% | Frontal |  |
| 2 | 0.250 | 74.4% | 0.182 | 81.1% | 0.132 | 86.8% | Parietal | Temporal |
| 3 | 0.352 | 68.1% | 0.275 | 75.4% | 0.199 | 83.1% | Parietal | Temporal |
| 4 | 0.372 | 72.1% | 0.257 | 73.2% | 0.187 | 73.9% | Frontal |  |
| 5 | 0.319 | 74.4% | 0.250 | 83.0% | 0.196 | 89.9% | Parietal |  |
| 6 | 0.383 | 69.1% | 0.322 | 77.0% | 0.249 | 82.7% | Frontal | SMA |
| 7 | 0.338 | 53.0% | 0.261 | 58.9% | 0.192 | 65.7% | Occipital | Temporal |
| 8 | 0.451 | 71.0% | 0.385 | 80.2% | 0.314 | 87.9% | Precentral | SMA |
| 9 | 0.448 | 68.3% | 0.399 | 77.8% | 0.324 | 85.2% | SMA |  |
| 10 | 0.342 | 63.3% | 0.279 | 69.2% | 0.212 | 74.3% | Temporal |  |
| 11 | 0.374 | 58.2% | 0.312 | 68.1% | 0.252 | 80.2% | Occipital |  |
| 12 | 0.498 | 77.7% | 0.416 | 86.1% | 0.314 | 92.3% | Precentral |  |
| 13 | 0.334 | 75.7% | 0.253 | 81.0% | 0.178 | 81.9% | Occipital |  |
| 14 | 0.287 | 79.9% | 0.174 | 84.8% | 0.109 | 82.7% | Occipital | Temporal |
| 15 | 0.397 | 66.9% | 0.310 | 72.3% | 0.236 | 82.9% | Frontal |  |
| 16 | 0.486 | 70.8% | 0.417 | 79.7% | 0.328 | 86.0% | Parietal | Postcentral |
| 17 | 0.367 | 47.8% | 0.311 | 55.3% | 0.255 | 62.4% | Temporal |  |
| 18 | 0.598 | 78.1% | 0.529 | 86.2% | 0.416 | 93.1% | Precentral |  |
| 19 | 0.588 | 68.8% | 0.543 | 78.5% | 0.457 | 87.2% | Postcentral |  |
| 20 | 0.463 | 51.6% | 0.424 | 59.1% | 0.365 | 68.2% | Temporal |  |
| 21 | 0.402 | 46.8% | 0.339 | 52.3% | 0.282 | 64.7% | *DRC |  |
| 22 | 0.430 | 71.7% | 0.330 | 75.7% | 0.245 | 81.3% | Frontal |  |
| 23 | 0.403 | 58.0% | 0.345 | 70.2% | 0.267 | 84.3% | SMA |  |
| 24 | 0.395 | 40.4% | 0.400 | 57.3% | 0.339 | 75.5% | *DRC |  |
| 25 | 0.367 | 46.8% | 0.276 | 54.0% | 0.202 | 66.6% | *DRC |  |
| 26 | 0.459 | 44.6% | 0.462 | 57.4% | 0.403 | 73.6% | *DRC |  |
| 27 | 0.499 | 57.4% | 0.450 | 67.3% | 0.356 | 73.3% | Frontal |  |
| 28 | 0.332 | 37.1% | 0.327 | 48.9% | 0.291 | 62.4% | *DRC |  |
| 29 | 0.500 | 55.4% | 0.474 | 67.1% | 0.408 | 76.8% | Postcentral | Parietal |
| 30 | 0.385 | 41.1% | 0.388 | 56.1% | 0.322 | 71.3% | Precentral |  |

*DRC indicates that the component does not reach a cortical target
